# The Effects of Higher Protein Intake on Muscle Mass and Clinical Outcomes in Critically Ill Cancer Patients: A Prespecified Per-Protocol Analysis

**DOI:** 10.3390/nu17172742

**Published:** 2025-08-24

**Authors:** Jerusa Marcia Toloi, Ana Carolina Gallo Laranja, Diogo Oliveira Toledo, Ricardo Esper Treml, Luiz Marcelo S. Malbouisson, William Manzanares, João Manoel Silva-Jr

**Affiliations:** 1 Division of Anesthesiology, Postgraduate Program of Anesthesiology, Surgical Sciences and Perioperative Medicine, Faculty of Medicine, University of São Paulo, Av. Dr. Arnaldo 455, Sao Paulo 05403-000, Brazil; malbouisson@gmail.com; 2 Department of Nutrition, Barretos Cancer Hospital, R. Antenor Duarte Vilela 1331, Barretos 14784-400, Brazil; aanacarolnutricionista@gmail.com; 3 Department of Nutrition, Hospital Israelita Albert Einstein, Av. Albert Einstein, 627/701, Sao Paulo 05652-900, Brazil; diogootoledo@gmail.com; 4 Department of Anesthesiology, Perioperative and Pain Medicine, School of Medicine, Stanford University, Palo Alto, CA 94305, USA; rictreml@stanford.edu; 5 Department of Critical Care, University Hospital of Universidad de la Republica (UDELAR), Av. Italia, Montevideo 11.600, Uruguay; wmanzanares@vera.com.uy; 6 Division of Critical Care, Department of Surgery, Ribeirão Preto Medical School, University of São Paulo, Av. Bandeirantes, 3900, Ribeirao Preto 14040-900, Brazil

**Keywords:** protein requirement, nutrition assessment, critical care, cancer, ultrasonography, quadriceps muscle

## Abstract

Background/Objectives: The optimal protein dose for critically ill cancer patients, especially for muscle mass preservation and survival, remains unclear. This study evaluated whether a higher protein intake, compared to usual intake, was associated with improved clinical outcomes in this population. Methods: This was a prospective analysis of critically ill adult cancer patients admitted to an oncological intensive care unit (ICU). Patients were initially assigned to receive protein prescriptions of either 1.5 or 2.0 g per kilogram per day (g/kg/day), but due to common limitations in achieving prescribed targets in this setting, a prespecified per-protocol analysis was conducted. After three days of exclusive nutritional therapy, patients were reclassified into two groups based on actual protein intake: >1.5 g/kg/day (higher intake group, IG) and ≤1.5 g/kg/day (usual intake group, CG). The primary outcome was muscle mass preservation, measured by quadriceps muscle thickness (QMT) via ultrasound on days 1, 7, and 14. Secondary outcomes included ICU survival, hospital and ICU length of stay, mechanical ventilation duration, dialysis requirement, and 60-day survival. Results: From June 2019 to September 2023, 117 patients were included. Following the planned interim analysis, the study was stopped after meeting the Pocock criterion for ICU survival (*p* = 0.0013). After reclassification, 68.4% (*n* = 80) were in the IG and 31.6% (*n* = 37) in the CG. ICU stay was similar (both medians 13 days), but the IG had shorter hospital stays [21.0 vs. 27.5 days, *p* = 0.020], less QMT loss, and improved ICU (HR = 0.31, 95% CI 0.15–0.64), hospital (HR = 0.43, CI 0.23–0.80), and 60-day survival (HR = 0.43, CI 0.23–0.80), along with shorter ventilation duration (HR = 0.54, CI 0.30–0.99). Conclusions: Higher protein intake (>1.5 g/kg/day) was associated with better muscle mass preservation and improved clinical outcomes in critically ill cancer patients.

## 1. Introduction

Advancements in diagnostic and therapeutic strategies have prolonged the survival of cancer patients, consequently increasing the number of intensive care unit (ICU) admissions in this population [1]. A Brazilian study reported that oncological patients account for approximately 20% of ICU bed occupancy, indicating that one in five ICU admissions involves a patient with cancer [2].

Critical illness, particularly when associated with cancer, triggers a systemic inflammatory response that accelerates muscle protein degradation and impairs synthesis. This leads to rapid muscle mass loss, with studies showing reductions of up to 20% within the first 10 days of ICU stay [3]. In cancer patients, muscle wasting is further exacerbated by tumor-related factors and chronic inflammation, which stimulate proteolysis via the ubiquitin–proteasome pathway and suppress anabolic responses [3,4].

Malnutrition in oncological ICU patients results from a combination of stress-induced catabolism, increased metabolic demands, and an insufficient nutritional supply [3]. This imbalance contributes to higher risks of complications, organ dysfunction, and mortality. Optimizing nutritional support—especially adequate protein provision—is therefore critical to mitigating these adverse effects and preserving skeletal muscle [5]. However, current guidelines lack robust recommendations for optimal protein dosing in critically ill cancer patients, due to a scarcity of high-quality interventional trials [6,7].

In this context, the present study aimed to evaluate whether higher protein intake is associated with better muscle mass preservation and improved clinical outcomes in critically ill cancer patients. To account for real-world variability in actual protein delivery, a prespecified per-protocol analysis was conducted based on achieved protein intake during the early phase of nutritional therapy. Our hypothesis is that critically ill cancer patients who receive higher protein intake will exhibit greater muscle mass preservation and improved survival compared to those receiving usual protein intakes.

## 2. Materials and Methods

### 2.1. Study Design

This was a prospective interventional study conducted in the intensive care unit (ICU) of a specialized cancer hospital in Brazil. Patients were initially assigned to receive either a standard or higher protein prescription. However, due to known limitations in achieving prescribed nutritional targets in critically ill patients, a prespecified per-protocol analysis was planned and conducted. Patients were reclassified based on their actual protein intake after three full days of exclusive nutritional therapy.

The study was approved by the local Research Ethics Committee (CAAE: 03452818.6.0000.5437) and registered in the Brazilian Clinical Trials Registry (ReBEC; UTN: U1111-1235-1979), a publicly accessible registry recognized by the World Health Organization (WHO) and the International Committee of Medical Journal Editors (ICMJE). Full details are available at https://ensaiosclinicos.gov.br (accessed on 20 August 2025). Written informed consent was obtained from all participants or their legal representatives.

### 2.2. Study Population

Eligible participants were adult patients (≥18 years), of both sexes, receiving exclusive nutritional therapy (enteral, parenteral, or both), with an anticipated ICU stay longer than 72 h. Mechanical ventilation was not an inclusion criterion; however, the need for ventilatory support was documented during the ICU stay as part of the standard clinical characterization of the participants. Exclusion criteria included patients with limitations of life-sustaining treatment, those who were pregnant or postpartum, and individuals with pre-existing acute or chronic kidney injury requiring dialysis prior to enrollment.

### 2.3. Trial Procedures

Participants were screened upon ICU admission using the Nutrition Risk Screening 2002 (NRS-2002), a score indicating nutritional risk [8]. Energy requirements were primarily determined through indirect calorimetry or, when unavailable, estimated using the ASPEN 2022 pocket formula [9]. Indirect calorimetry was typically performed within the first 48 h after ICU admission once patients were hemodynamically stable. Caloric targets were reassessed daily. Protein requirements were calculated by multiplying the target protein allocation—1.5 g/kg/day for the control group or 2.0 g/kg/day for the intervention group—by the patient’s body weight (the latter used for patients with BMI > 30 kg/m^2^, using the formula: 25 × height^2^).

This intentional difference in prescribed doses was designed to account for known discrepancies between prescribed and administered protein in critically ill patients. By targeting 2.0 g/kg/day in the intervention group and 1.5 g/kg/day in the control group, the protocol anticipated a natural separation based on actual intake: patients receiving ≥1.5 g/kg/day were later allocated to the higher intake group, and those receiving <1.5 g/kg/day to the usual intake group.

Once the caloric target was achieved, protein intake was gradually increased according to group allocation and patient tolerance. Patients were followed until ICU discharge, transition to oral intake, or death. On Day 3, a prespecified per-protocol analysis was performed: actual protein intake over the prior 72 h was assessed, and patients were reclassified into two groups based on intake: ≥1.5 g/kg/day or <1.5 g/kg/day. This pragmatic approach allowed assessment of outcomes based on real protein exposure, reflecting clinical practice conditions where prescribed targets are often unmet due to interruptions, intolerance, or clinical instability.

Muscle mass was assessed by quadriceps muscle thickness (QMT) using a bedside portable ultrasound device (GE LOGIQ E, General Electric Healthcare Technologies, Chicago, IL, USA) with a multifrequency linear transducer (L4–12t) in B-mode. Evaluations were performed on Days 1, 7, and 14, with the patient in the supine position and knee extended. Two measurement sites were marked along the anterior thigh: (a) midpoint (½) and (b) distal third (⅔) between the anterosuperior iliac crest and the upper patella. QMT was defined as the distance between the upper border of the femur and the lower border of the superficial fascia of the rectus femoris. Measurements were repeated three times at each site, both with and without transducer compression, and the average was used for analysis [10].

All patients received standard ICU care, including mechanical ventilation, vasopressor support, renal replacement therapy, and antimicrobials, based on clinical indications. These cointerventions were equally available to all participants, minimizing potential confounding.

### 2.4. Nutritional Allocation and Blinding

At study initiation, patients were prospectively assigned to receive one of two nutritional strategies: a higher protein prescription of 2.0 g/kg/day or a usual prescription of 1.5 g/kg/day. Allocation was determined using adaptive algorithms within the Research Electronic Data Capture (REDCap) platform to maintain group balance based on predefined criteria (sex, age ≥ 60 years, nutritional risk status using NRS-2002, and admission type). Although this process ensured structured allocation, actual protein intake was frequently influenced by clinical limitations common in intensive care settings, such as feeding interruptions or intolerance.

Due to the nature of the intervention, full blinding was not feasible. The clinical team was aware of group assignments to ensure proper nutritional prescription and monitoring. However, patients and outcome assessors, including the statistician, were blinded to group classification. To address variability in real-world protein delivery and preserve analytic rigor, a prespecified per-protocol analysis was conducted based on actual protein intake after three days of exclusive nutritional therapy.

### 2.5. Outcomes

The primary outcome of the study was muscle mass preservation, assessed by changes in quadriceps muscle thickness (QMT) measured via ultrasound. Measurements were taken at two predefined anatomical points—½ and ⅔ of the distance between the anterosuperior iliac crest and the upper edge of the patella—with and without transducer compression. QMT was evaluated on Days 1 (D1), 7 (D7), and 14 (D14) to capture the critical period of muscle mass alterations during ICU stay. These time points were selected based on previous studies by our group, which demonstrated significant muscle loss after the first week of critical illness [11].

A prespecified secondary outcome was ICU survival, which was prioritized for interim analysis. Other secondary outcomes included the duration of mechanical ventilation, need for dialysis after seven days of inclusion, ICU and hospital length of stay, hospital survival, and 60-day survival. All outcomes were analyzed in relation to the actual protein intake observed during the first three days of exclusive nutritional therapy.

### 2.6. Statistical Analysis

The sample size was estimated based on the findings of Weijs et al. [12], who reported a mortality rate of 8.8% in patients receiving ≥1.2 g/kg/day of protein versus 22% in those receiving <0.8 g/kg/day. Using Fisher’s exact test with a 5% significance level and 80% power, the required sample size was 226 patients (113 per group), as calculated with G*Power version 3.1.9.4 (Heinrich Heine University, Düsseldorf, Germany) [13]. An interim analysis was prespecified once 50% of the estimated sample had been included (*n* = 113), with ICU survival as the primary endpoint for this analysis. The trial was stopped early after meeting the predefined Pocock criterion (*p* < 0.025).

Due to well-recognized challenges in delivering the prescribed protein dose in critically ill patients, a prespecified per-protocol analysis was planned to reflect actual protein intake. Three days after initiating exclusive nutritional therapy, patients were reclassified into two groups: those who received ≥1.5 g/kg/day (higher intake group, IG) and those who received <1.5 g/kg/day (usual intake group, CG). To support the accuracy of this classification, a K-means clustering algorithm was applied as a supplementary method to confirm grouping based on intake similarity (see Supplementary Figure S1).

Normality was assessed using the Kolmogorov–Smirnov test. Continuous variables were compared using t-tests or Mann–Whitney U tests, as appropriate. Categorical variables were compared using the chi-square or Fisher’s exact test. A general linear model was used to compare QMT changes over time, with Bonferroni correction applied for multiple comparisons. Multivariate analyses adjusted outcomes for nutritional risk (NRS-2002) to reduce residual confounding.

Survival analyses were performed using the Kaplan–Meier method and log-rank tests. Cox proportional hazards models were used to adjust for baseline nutritional risk (NRS-2002), providing hazard ratios (HRs) with 95% confidence intervals (CIs) for ICU, hospital, and 60-day survival, as well as duration of mechanical ventilation.

Data were managed in REDCap^®^ version 11.1.18 [14] and analyzed using IBM SPSS Statistics^®^ version 29.0.2.0 (Chicago, IL, USA). A *p*-value < 0.05 was considered statistically significant.

## 3. Results

Between June 2019 and September 2023, a total of 427 patients were assessed for eligibility. Of these, 284 were excluded due to reasons such as not meeting inclusion criteria, declining to participate, or developing acute kidney injury prior to enrollment. A total of 117 patients were included in the analysis. An interim analysis conducted after enrolling 50% of the planned sample met the Pocock stopping criterion for ICU survival (*p* = 0.0013), leading to early termination of the trial.

**Figure 1:**
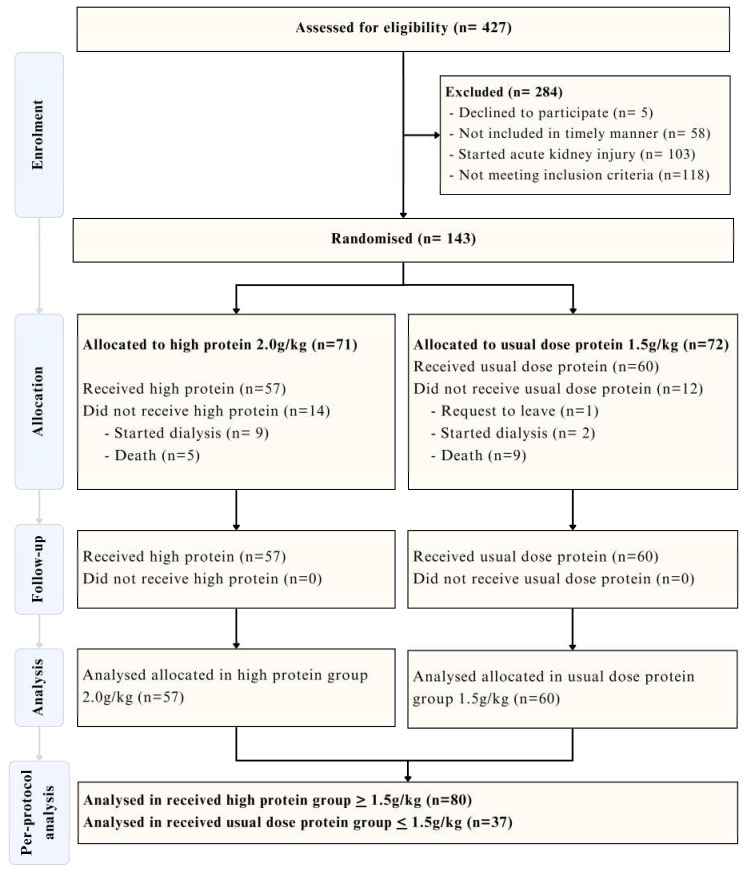
Flowchart of patient enrollment, allocation, follow-up, and analysis (per-protocol analysis). Symbols: *n*, Number of patients. Abbreviations: g/kg, grams per kilogram. Terms: high protein group, patients who received ≥1.5 g/kg/day; usual protein group: patients who received <1.5 g/kg/day.

Although patients were originally assigned to receive either 1.5 or 2.0 g/kg/day of protein, the actual amount administered was frequently influenced by clinical limitations, including feeding interruptions and gastrointestinal intolerance. Therefore, participants were reclassified based on the actual mean protein intake received by Day 3 of nutritional therapy. A total of 80 patients (68.4%) received ≥1.5 g/kg/day and were included in the higher protein group (IG), while 37 patients (31.6%) received <1.5 g/kg/day and were included in the usual intake group (CG) (Figure 1).

As illustrated in Supplementary Figure S1, despite the original prescription targets, the actual protein intake in the intervention group approximated 1.5 g/kg/day, underscoring the practical difficulties in achieving higher protein doses in critically ill patients.

Baseline characteristics were generally well balanced between groups. There were no significant differences in age, sex, ICU admission timing, or nutritional therapy initiation. A slightly higher proportion of surgical patients was observed in the high-protein group. Severity scores (SAPS 3 and SOFA), nutritional risk (NRS-2002), and body mass index (BMI) were comparable across groups. As expected, protein intake was higher in the IG group. The distribution of vasopressor use, physiotherapy modalities, and kidney function markers (creatinine and urea) also supported clinical comparability between groups (Table 1). A detailed breakdown of oncologic subtypes and ICU admission characteristics is provided in Supplementary Tables S1 and S2.

Enteral nutrition was administered to 91.9% of patients in the usual intake group (CG), 82.5% of patients in the higher protein group (IG), and 85.5% of the overall population, indicating that most patients had enteral access during the intervention period. Parenteral nutrition was used in 5.4%, 12.5%, and 10.3% of patients, respectively, and mixed (enteral plus parenteral) nutrition was provided in 2.7%, 5.0%, and 4.3% of patients across groups (*p* = 0.404). Protein supplementation included intravenous amino acids in 0.0% of the CG, 19.1% of the IG, and 14.1% of the total population. Casein was administered to 37.5% of the CG, 11.8% of the IG, and 18.5% overall. Whey protein supplementation was received by 52.5% of the CG, 69.1% of the IG, and 63.9% of all patients (*p* = 0.004).

**Figure 2:**
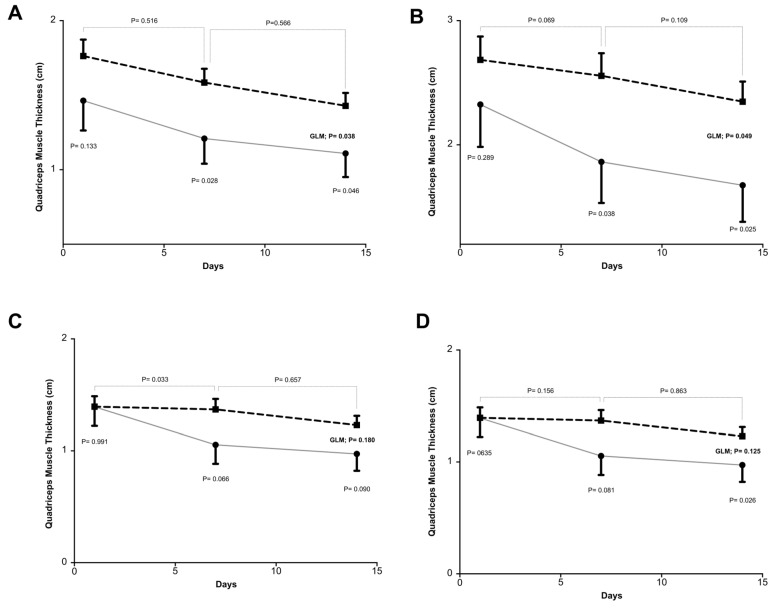
Comparison between groups regarding quadriceps muscle thickness in a multivariate analysis during the 14-day follow-up after study inclusion. The dashed line represents the intervention group (*n* = 80), and the solid line represents the control group (*n* = 37), with respective means and standard deviations. Muscle thickness measurements were taken on the first, seventh, and fourteenth days of follow-up. (**A**,**B**) represent measurements taken at the mid-thigh, with and without transducer compression, respectively. (**C**,**D**) represent measurements taken at the distal third of the thigh, with and without transducer compression, respectively. GLM, generalized linear model.

Quadriceps muscle thickness (QMT) decreased on average by 13.2% after one week (*n* = 91) and 30.7% after two weeks (*n* = 43). The most representative measurement, taken at the mid-thigh without compression, showed an average loss of 41% over two weeks, equivalent to −0.41 cm in absolute terms. When stratified by group, the CG exhibited significantly greater muscle loss than the IG. In the first week, the CG lost an average of −0.22 cm (22%) versus −0.09 cm (9%) in the IG. By the second week, losses were −0.49 cm (49%) in the CG and −0.26 cm (26%) in the IG (Figure 2).

After adjustment for nutritional risk (NRS-2002), clinical outcomes favored the higher protein group. The IG showed a shorter hospital length of stay [21.0 days (IQR 15.2–29.5) vs. 27.5 days (IQR 17.5–42.0), *p* = 0.020]. The incidence of dialysis was lower in the IG (8.8% vs. 18.9%), with a relative risk (RR) of 0.41, suggesting a potential renal benefit. ICU survival was significantly higher in the IG, with 77.5% discharged alive compared to 56.8% in the CG (*p* = 0.029), corresponding to an RR of 2.55 for ICU mortality in the CG. However, no statistically significant differences were observed in 60-day outcomes, including survival status and cancer progression (Table 2). Sensitivity analyses using the ITT population are presented in Supplementary Table S3, showing results consistent with the per-protocol analysis.

**Figure 3:**
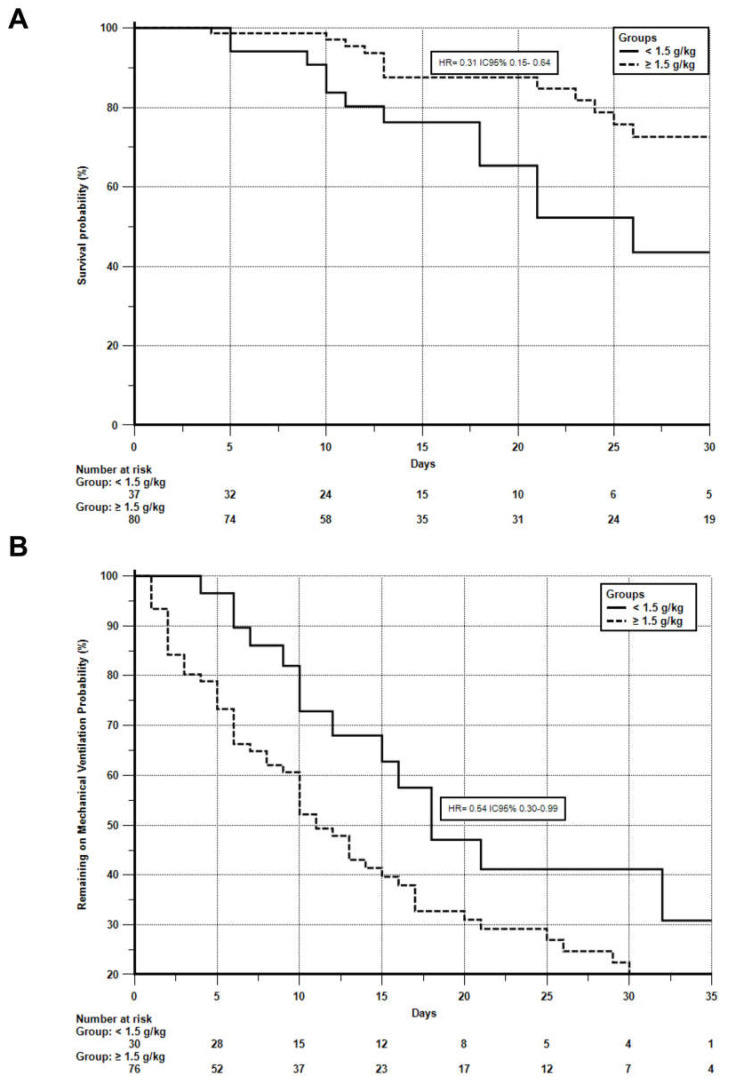
Kaplan–Meier curves comparing ICU survival and mechanical ventilation duration, adjusted for Nutritional Risk Screening (NRS-2002). Legends: The dashed line represents the intervention group, and the solid line represents the control group. Image (**A**) refers to ICU survival. Image (**B**) refers to the duration of mechanical ventilation (*n* = 106). The adjustment was made according to the Cox model. HR, hazard ratio (relative risk of the event occurrence over time). NRS-2002, Nutrition Risk Screening 2002.

Survival analysis adjusted for nutritional risk (NRS-2002) showed that ICU survival was significantly higher in the high-protein group (IG) compared to the usual intake group (CG), with a *p*-value of 0.0013 [hazard ratio (HR) = 0.31, 95% confidence interval (CI): 0.15–0.64]. Additionally, patients in the IG experienced a shorter duration of mechanical ventilation (*n* = 106, 90.6%), with a statistically significant difference (*p* = 0.0370; HR = 0.54, 95% CI: 0.30–0.99) (Figure 3).

**Figure 4:**
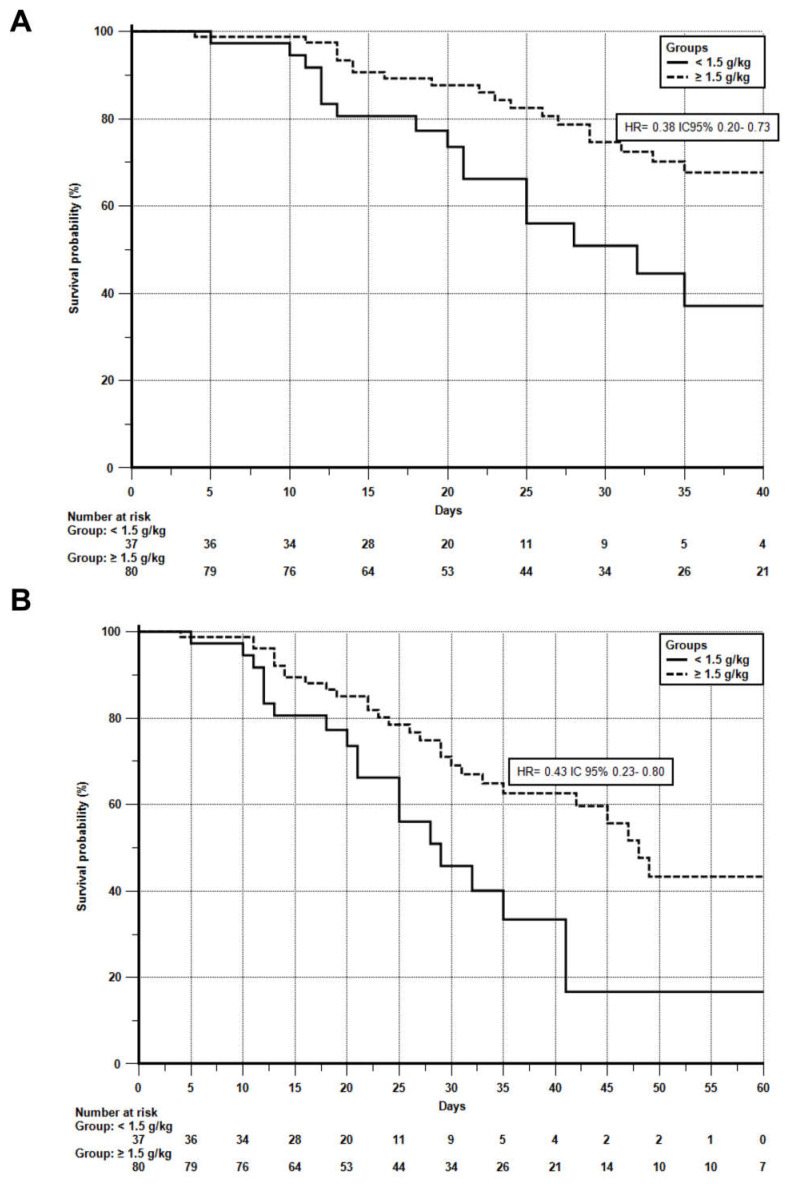
Kaplan–Meier curves comparing hospital survival and 60-day survival after inclusion in the study, adjusted for Nutritional Risk Screening (NRS-2002). The dashed line represents the intervention group and the solid line the control group. Image (**A**) relates to hospital survival. Image (**B**) relates to 60-day survival. Adjustment was made according to the Cox model. HR, hazard ratio (relative risk of event occurrence over time). NRS-2002, Nutrition Risk Screening 2002.

Similarly, hospital survival was greater in the high-protein group (*p* = 0.0033; HR = 0.43, 95% CI: 0.23–0.80), as was 60-day survival (*p* = 0.0072; HR = 0.43, 95% CI: 0.23–0.80) (Figure 4).

## 4. Discussion

In this prespecified per-protocol analysis, administering a higher protein dose (≥1.5 g/kg/day) to critically ill cancer patients in the ICU was associated with better muscle mass preservation and improved survival outcomes. The findings from the per-protocol analysis indicate that patients who received greater protein intake experienced less muscle loss, as measured by quadriceps muscle thickness, and had better ICU, hospital, and 60-day survival rates. Additionally, the group with higher protein intake demonstrated a shorter duration of mechanical ventilation and a reduced length of hospital stay, suggesting that optimizing protein dosing in this population is crucial for enhancing clinical outcomes. These results provide valuable evidence supporting the importance of nutritional support, especially protein intake, in the care of critically ill cancer patients.

To fully understand the benefits of higher protein dosing in critically ill patients, it is essential to relate our findings to existing clinical practice challenges and outcomes observed in prior studies. It is well-established that in clinical practice, the prescribed protein intake often does not correspond to the amount actually delivered, leading to many patients not receiving the guideline-recommended targets, particularly during the first week of ICU admission [6,9]. It is important to consider the clinical challenges often faced in meeting nutritional targets. In ICU settings, the prescribed protein intake often differs from the actual intake due to frequent interruptions, such as patient instability, procedures, or intolerance. This justified our prespecified decision to conduct a per-protocol analysis, which was planned to better reflect real-world protein delivery and its clinical implications, particularly in ICU settings with frequent interruptions to nutritional therapy.

In our study, we found that the average protein intake by patients from the fourth day of ICU admission was 1.49 g/kg/day, with an impressive infusion rate of 96.2%. This result is particularly noteworthy when compared to the literature, where similar studies often report lower infusion rates and difficulties in achieving protein targets [15,16,17]. The high infusion rate in our study highlights the effectiveness of our nutritional strategy and its adherence to protocol, which is a key factor in achieving the desired outcomes.

Moreover, the median time to reach nutritional intake (both caloric and protein) was 4 days, with a steady progression of 25% of the targets per day. This strategy aligns with the phases of the metabolic response to critical illness, as recommended and supported by recent research [18]. By following these guidelines, our study not only met but sustained adequate protein intake, which is vital for maintaining muscle mass and improving survival outcomes. It is noteworthy that over 50% of patients received a daily protein dose exceeding 1.5 g/kg by day 3 of ICU admission. This decision was made based on the patients’ nutritional needs, as determined by their clinical condition and baseline nutritional risk, assessed through the NRS-2002. Although critically ill patients are often at risk of complications such as gastrointestinal intolerance or metabolic disturbances, higher protein intake is essential to prevent muscle wasting and support recovery in patients who are not severely compromised in their metabolic and organ function. Importantly, the administration of higher protein doses was carefully monitored and adjusted according to the patients’ tolerance and overall condition. Despite relatively moderate SAPS III and SOFA scores, extended ICU and hospital stays are expected in oncologic ICU populations due to delayed recovery, immunosuppression-related complications, and the need to resume or complete oncologic treatments post-critical illness.

In this study, the patients who received higher doses of protein were those with a lower likelihood of intolerance, as assessed by their clinical course and absence of contraindications to nutritional therapy. Furthermore, the safety and efficacy of this approach were validated through the study’s secondary outcomes, showing no significant increase in adverse effects such as dialysis requirements or prolonged ICU stay in the higher protein group. The decision to administer a protein dose exceeding 1.5 g/kg/day by day 3 was based on the premise that early and adequate protein provision can mitigate the catabolic effects of critical illness, particularly in cancer patients. To assess the robustness of our findings, sensitivity analyses using the intention-to-treat population were performed and are presented in Supplementary Table S3. Although the trial was stopped early, which may overestimate treatment effects, sensitivity analyses using the ITT population showed similar trends in direction, despite the fact statistical significance was not reached due to reduced power.

These findings are further validated by comparisons with earlier studies [19], which highlighted the benefits of higher protein doses in critically ill patients. However, our study’s focus on critically ill cancer patients allowed us to provide more specialized and targeted evidence for this vulnerable population. Additionally, using ultrasonography to assess quadriceps muscle thickness offered a more precise evaluation of muscle preservation, which is especially relevant in an oncological ICU setting where muscle wasting can have severe consequences. A meta-analysis published in 2023 reinforces that ultrasound is the most prevalent tool used in the ICU for evaluating muscle mass [20]. Integrating these findings with the existing literature reinforces the importance of personalized nutritional care in the ICU, particularly for oncological patients [21].

The positive correlation between higher protein intake and muscle mass preservation aligns with Azevedo et al.’s study [22], which emphasized the combined role of exercise and nutrition. Notably, our study showed that increased protein intake alone, even without differences in motor intervention between groups, can significantly preserve muscle mass and improve survival, particularly in patient populations where exercise interventions may be difficult or impractical. This conclusion is consistent with the meta-analysis by van Ruijven et al. [7], which found that protein provision exceeding 1.2 g/kg was associated with improved nitrogen balance, muscle mass preservation, and potentially better 60-day mortality outcomes. While our findings highlight the benefits of higher protein intake for muscle preservation and short-term survival, they did not reveal an impact on longer-term outcomes. This suggests that the advantages of increased protein intake may depend on factors such as underlying health conditions and the timing of administration, necessitating further research.

During follow-up, our patients exhibited a modest increase in serum urea levels (CG 75 [47.8–105.1] vs. IG 66.9 [49.5–94.7], *p* = 0.462) and creatinine levels (CG 0.60 [0.39–1.02] vs. IG 0.55 [0.38–0.78], *p* = 0.231), without an increased need for dialysis (CG 18.9% vs. IG 8.8%, *p* = 0.129). This outcome is consistent with a clinical study [23] involving a similar patient number and intervention duration as ours. Conversely, in the reanalysis of the EFFORT Protein Trial RCT [24], a twofold increase in urea levels was observed in patients who received higher protein loads, which was linked to a higher risk of 30-day mortality (HR = 1.34, 95% CI 1.21–1.48). Unlike the EFFORT trial [25], our study focused specifically on critically ill cancer patients, which allowed us to avoid including patients with pre-existing renal injury and to conduct a more rigorous per-protocol analysis, ensuring that actual protein intake was accurately reflected in the results. Our findings also differ from EFFORT [25] and other recent trials, which tested higher protein targets (>2 g/kg/day) and included heterogeneous ICU populations. In contrast, our patients were exclusively oncological, moderately ill (lower SAPS/SOFA), and received an intermediate protein target (~1.5 g/kg/day). These differences may partly explain the distinct outcomes observed.

Similarly, while the PRECISe trial [26] provided valuable insights into protein supplementation in critically ill patients, it did not specifically address the unique needs of oncological patients or thoroughly investigate the effects of protein intake on muscle mass preservation using direct methods, such as quadriceps muscle thickness (QMT) measurements. By focusing on critically ill cancer patients and employing precise and direct assessments of muscle mass, our study addresses these gaps. This approach strengthens the evidence for tailored nutritional strategies in this specific patient population, offering more targeted insights than previous research.

Notably, while the prescribed doses in trials such as the PRECISe [26] and EFFORT [25] trials exceeded 2 g/kg/day, actual protein intake in these studies (e.g., 1.6 g/kg/day in EFFORT) was similar to the achieved intake in our study, reinforcing the practical challenge of reaching target doses in critically ill patients. Although our findings demonstrate that this moderate dose was effective in preserving muscle mass and improving survival in critically ill cancer patients, the discrepancy between prescribed and actual intake across studies suggests that the optimal protein dose may lie between the typical range of less than 1.3 g/kg/day and up to 2 g/kg/day. This perspective is further supported by the findings in the systematic review [27], which reported positive outcomes with a protein intake of approximately 1.5 g/kg/day, similar to our findings. However, while our study focused on critically ill cancer patients, the systematic review [27] explored a broader ICU population, highlighting the need for additional research to determine the most effective protein dosing strategy across different critically ill populations, accounting for the specific needs of each patient group.

The strengths of this study include a well-conducted randomization process, which minimizes selection bias, and the fact that both protein targets are within the recommended range, made possible by the prior per-protocol goal that even in the control group considered protein values greater than 1.5 g/kg/day, ensuring that both groups received high-quality care. Moreover, conducting the study in an ICU specialized in oncological patients reflects the specific care required for this population. Additionally, to the best of our knowledge, this is the first study specifically designed for this population. A key point highlighted by this study is the necessity for personalized nutritional care for ICU patients, given the wide variation in patient profiles and the lack of evidence-based recommendations for this diverse population. This study fills a gap in the literature by focusing on a specific population of ICU oncology patients, whose incidence is steadily increasing. Consequently, this study contributes to clinical practice by providing valuable insights into optimal nutritional strategies in this context.

Nevertheless, several limitations of this study should be acknowledged. One notable limitation is its focus on an oncological ICU population, which may restrict the generalizability of the findings to broader ICU settings. Additionally, the challenges posed by the COVID-19 pandemic during the study period likely influenced patient recruitment. The 5-year recruitment period reflects the specificity of our inclusion criteria and was impacted by the COVID-19 pandemic, which interrupted ICU admissions and elective procedures for approximately 24 months. However, these unique and stressful healthcare conditions also provided an opportunity to evaluate the intervention under circumstances that may enhance its practical relevance.

While the study was conducted in a single center and was single-blinded, these limitations are mitigated by the specialized nature of the oncological ICU and the rigorous study design. The single-center setting allowed for a controlled environment with consistent care protocols and nutritional management, ensuring the reliable assessment of the effects of higher protein dosing on clinical outcomes. Although the intervention was not blinded, the standardized application of care minimized potential biases related to the open-label nature of the study.

One important limitation is the absence of an intention-to-treat analysis, which limits the inference of causality. However, the per-protocol approach was prespecified to reflect actual protein delivery, and group reclassification was based on measured intake after three days of exclusive nutritional therapy, minimizing misclassification.

The variability introduced by the use of different enteral and parenteral nutrition formulas reflects real-world clinical practice, where individualized nutrition plans are routinely tailored to patient needs. This variability strengthens the external validity of the findings by simulating typical ICU conditions. Lastly, although the study did not evaluate long-term functional outcomes in critically ill patients, the robust short-term data provide valuable insights to inform immediate clinical decision-making. Future multicenter and adequately powered studies, with robust adjustment for confounders and assessment of long-term functional outcomes, are warranted to confirm the role of higher protein dosing in critically ill cancer patients.

## 5. Conclusions

This prespecified per-protocol analysis demonstrated that a higher protein intake strategy (>1.5 g/kg/day) was associated with better muscle mass preservation and improved short-term survival in critically ill cancer patients, without increasing the risk of renal complications. These findings support the importance of individualized protein dosing in this vulnerable population. Nonetheless, well-designed, adequately powered multicenter clinical trials are warranted to confirm these findings and to assess long-term clinical and functional outcomes.

## Figures and Tables

**Table 1:** Characteristics of patients by protein intake group.

Characteristics	Usual Dose (*n* = 37)	High Protein (*n* = 80)	Variance (IC 95%)
Sex, *n* (%)			
Female	14 (37.8)	42 (52.5)	14.7 (−14.5–39.3)
Male	23 (62.2)	38 (47.5)	14.7 (−10.7–37.1)
Age (year), median [IQR]	63 [50.5–67.5]	60.5 [47.5–68.5]	−1.0 (−6.0–3.0)
Age rating, *n* (%)			
Elderly (≥60 years)	19 (51.4)	39 (48.8)	2.6 (−23.0–27.7)
Adult (≤59 years)	18 (48.6)	41 (51.2)	2.6 (−23.2–27.9)
Time from ICU admission to randomization (days), median [IQR]	1.0 [1.0–2.25]	1.0 [1.0–2.0]	0.0 (−1.0–0.0)
Admission category, *n* (%)			
Medical	19 (51.4)	32 (40.0)	11.4 (−15.5–36.6)
Surgical	18 (48.6)	48 (60.0)	11.4 (−14.1–35.6)
Oncology medical specialty, *n* (%)			
Neurologist	16 (43.2)	32 (40.0)	3.2 (−23.5–30.8)
Chest	6 (16.2)	9 (11.3)	4.9 (−30.1–45.7)
Proctology	2 (5.4)	10 (12.5)	7.1 (−57.6–38.2)
Gastroenterology	5 (13.5)	6 (7.5)	6.0 (−35.6–49.9)
Hematology	6 (16.2)	5 (6.3)	9.9 (−35.9–50.0)
Gynecology	1 (2.7)	7 (8.8)	6.1 (−72.0–43.1)
Urology	1 (2.7)	4 (5.0)	2.3 (−75.6–51.2)
Head and neck	0 (0.0)	5 (6.3)	6.3 (−73.3–50.1)
Melanoma/sarcoma	0 (0.0)	2 (2.5)	2.5 (−76.9–67.4)
SAPS 3 (point), median [IQR]	47.0 [36.0–65.0]	46.0 [35.5–57.0]	−3.0 (−9.0–4.0)
SOFA (point), median [IQR]	4.0 [2.0–7.0]	4.5 [2.0–7.5]	0.0 (−1.0–1.0)
NRS-2002 (point), median [IQR]	2.0 [1.75–3.25]	2.0 [2.0–4.0]	0.0 (0.0–1.0)
BMI, median [IQR]	25.8 [23.7–28.2]	25.6 [22.7–29.5]	−0.15 (−28.7–28.4)
Unintentional weight loss before admission to hospital, *n* (%)	8 (21.6)	31 (38.8)	17.1(−20.3–34.5)
Resting Energy Expenditure by indirect calorimetry (kcal/day), median [IQR]	1540 [1297.7–1881.0]	1552.0 [1303.5–1809.7]	22.0 (−212.0–251.0)
Follow-up during intensive care			
Use of vasopressor, *n* (%)	15 (40.5)	23 (28.7)	11.8 (−0.2–23.7)
Physical Therapy, *n* (%)			
Passive	27 (73.0)	56 (70.0)	3.0 (−8.2–14.2)
Active	8 (21.4)	14 (17.5)	3.9 (−4.9–12.7)
Resistive	2 (5.4)	10 (12.5)	−7.1 (−14.6–0.4)
Calories (kcal/day), median [IQR]	1526.7 [1151.4–1783.9]	1575.4 [1440.0–1743.0]	110.1 (−25.0–262.0)
Proteins (g/kg/day), median [IQR]	1.13 [0.93–1.22]	1.63 [1.5–1.85]	0.6 (0.5–0.7)
Creatinine (mg/dL), median [IQR]	0.60 [0.39–1.02]	0.55 [0.38–0.78]	−0.07 (−0.2–0.05)
Urea (mg/dL), median [IQR]	75.0 [47.8–105.1]	66.9 [49.5–94.7]	−4.85 (−19.9–9.3)

Abbreviations: *n*, number of patients; IQR, interquartile range; IC 95%, confidence interval; ≥, more or equal; ≤, less or equal; ICU, intensive care unit; SAPS 3, Simplified Acute Physiology Score 3; SOFA, Sequential Organ Failure Assessment; NRS-2002, Nutrition Risk Screening 2002; BMI, body mass index; mg, milligram; dL, deciliter. A chi-square test was used for categorical variables; the Mann–Whitney test was used for continuous variables.

**Table 2:** Clinical outcomes; analysis adjusted for nutritional risk screening (NRS-2002).

	Usual Dose (*n* = 37)	High Protein (*n* = 80)	*p* Value *	Relative Risk (IC 95%) *
Duration of ICU stay (days), median [IQR]	13 [8–21]	13 [10–28]	0.134	0.97 (0.9–1.0)
Dialysis, *n* (%)	7 (18.9)	7 (8.8)	0.129	0.41 (0.13–1.30)
Duration of Hospital stay (days), median [IQR]	27.5 [17.5–42.0]	21.0 [15.2–29.5]	0.020	1.03 (1.0–1.1)
MRC, median [IQR]	2.0 [1.0–5.75]	16 [2.5–46.5]	0.050	0.97 (0.94–1.00)
ECOG D7, median [IQR]	4.0 [3.0–4.0]	4.0 [3.0–4.0]	0.682	1.09 (0.72–1.64)
ECOG D14, median [IQR]	3.0 [3.0–4.0]	3.0 [3.0–4.0]	0.753	1.12 (0.56–2.23)
ICU outcome, *n* (%)			0.029	
Discharged	21 (56.8)	62 (77.5)		REFERENCE
Died	16 (43.2)	18 (22.5)		2.55 (1.09–5.93)
60-day outcome, *n* (%)				
Alive without cancer	8 (21.6)	28 (35.0)	0.323	REFERENCE
Alive with cancer	10 (27.0)	20 (25.0)	0.805	1.13 (0.43–2.98)
Dead	19 (51.4)	32 (40.0)	0.170	2.01 (0.74–5.49)

Abbreviations: *n*, number of patients; IQR, interquartile range; IC95% confidence interval; MRC, Medical Research Council; ECOG, Eastern Cooperative Oncology Group; ICU, intensive care unit; D7, day seven; D14, day fourteen. * Multivariate analysis adjusted for NRS-2002 (Nutrition Risk Screening 2002). The relative risk refers to the control group.

## Data Availability

The data presented in this study are available on request from the corresponding authors. The data are not publicly available due to privacy reasons.

## Supplementary Materials

The following supporting information can be downloaded at: https://www.mdpi.com/article/10.3390/nu17172742/s1, Figure S1: Comparison of Protein Intake Allocation Before and After Per Protocol 463 Analysis Using the K-means Method; Table S1: Baseline Characteristics by Oncologic Subtype and ICU Admission Context; Table S2: Protein Supplementation by Group with *p*-value; Table S3: Sensitivity Analyses Using Intention-to-Treat Population.
