# Supplementary material for: The Effects of Higher Protein Intake on Muscle Mass and Clinical Outcomes in Critically Ill Cancer Patients: A Prespecified Per-Protocol Analysis
Source: Nutrients. 2025 Aug 24;17(17):2742. doi: 10.3390/nu17172742 (PMC12429859; doi:10.3390/nu17172742)
Supplement: Supplementary file 1 [file nutrients-17-02742-s001.zip › nutrients-3823619-supplementary.pdf]

Supplementary

Supplementary Figure S1. Comparison of Protein Intake Allocation Before and After Per Protocol Analysis Using the K-means Method.

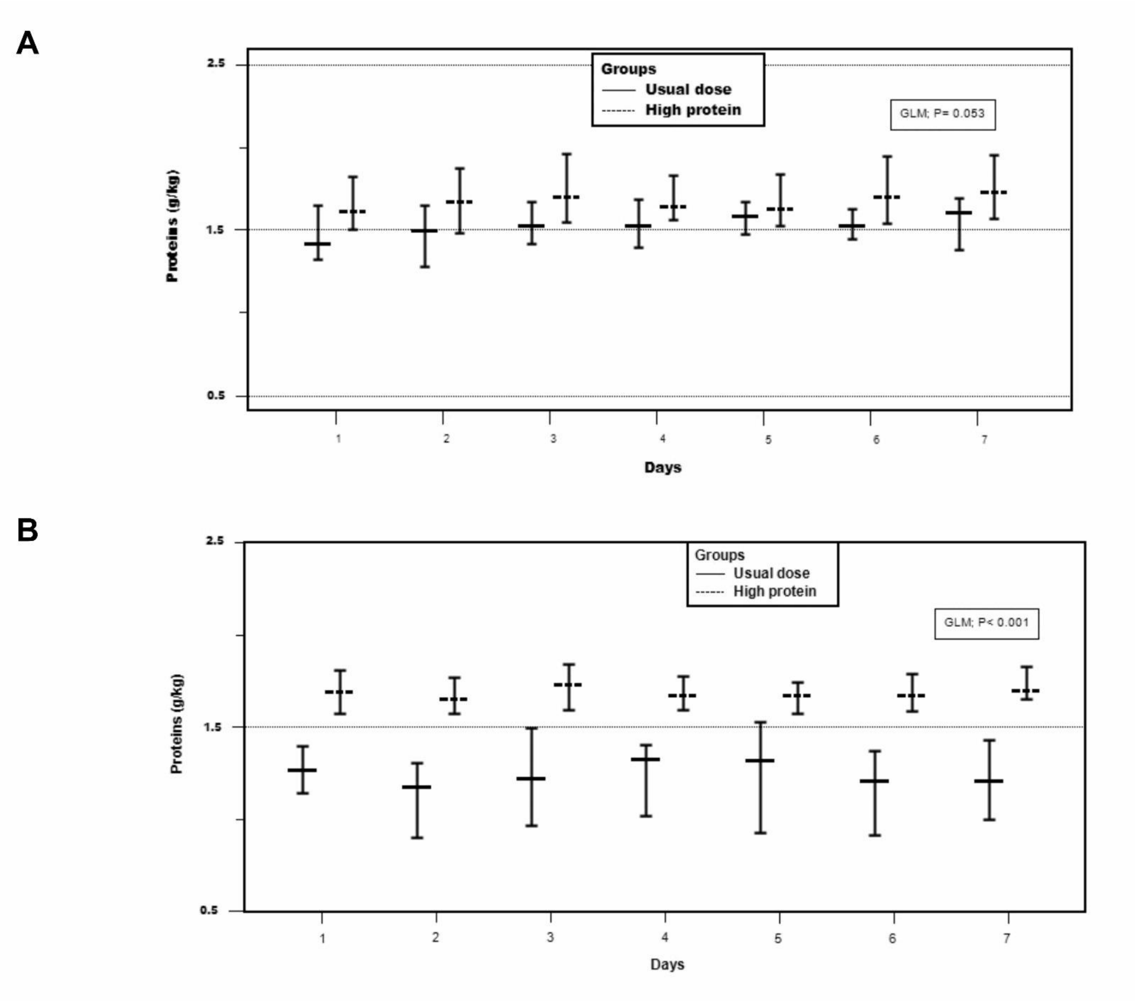

Legends: Figure A: Displays protein intake allocation for the entire population before the per protocol analysis, with the Control Group (CG) represented by a solid line and the Intervention Group (IG) represented by a dashed line. The data points reflect the average daily protein intake (g/kg/day) for each group over the course of 7 days.

Figure B: Depicts protein intake allocation after per protocol analysis, focusing on patients who adhered to the nutritional intervention as prescribed. The dashed line represents patients in the IG receiving more than 1.5 g/kg/day, while the solid line represents patients in the CG receiving less than or equal to 1.5 g/kg/day.

GLM (General Linear Model): Used to compare protein intake differences between groups over time. Statistical significance is indicated in both figures (GLM p-value displayed).

K-means: K-means Clustering Method was used to classify patients based on their protein intake into two distinct clusters.

Cluster 1: Patients receiving a high protein intake, represented by a specific dashed line pattern.

Cluster 2: Patients receiving usual protein intake, represented by a solid line.

Abbreviations: G: Intervention Group; CG: Control Group; g/kg/day: Grams of protein per kilogram per day.

Supplementary Table S1. Baseline Characteristics by Oncologic Subtype and ICU Admission Context

Characteristic	Usual Dose (≤1.5 g/kg/day) (n=37)	High Protein (>1.5 g/kg/day) (n=80)
Tumor location – n (%)		
CNS tumors	14 (37.8%)	26 (32.5%)
Head and neck	6 (16.2%)	11 (13.8%)
Gastrointestinal	5 (13.5%)	17 (21.3%)
Hematologic	7 (18.9%)	18 (22.5%)
Other	5 (13.5%)	8 (10.0%)
Treatment intent – n (%)		
Curative	17 (45.9%)	41 (51.3%)
Palliative	20 (54.1%)	39 (48.7%)
ICU admission type – n (%)		
Elective	11 (29.7%)	23 (28.8%)
Emergency	26 (70.3%)	57 (71.2%)
Reason for ICU admission – n (%)		
Postoperative	17 (45.9%)	35 (43.8%)
Sepsis / Infection	10 (27.0%)	22 (27.5%)
Oncologic therapy complication	6 (16.2%)	15 (18.8%)
Other (e.g., bleeding, airway)	4 (10.8%)	8 (10.0%)

Legend: CNS = Central Nervous System; ICU = Intensive Care Unit; n (%) = number and percentage of patients in each group. Usual Dose = protein intake ≤1.5 g/kg/day; High Protein = protein intake >1.5 g/kg/day. Treatment intent: Curative = aimed at cure; Palliative = aimed at symptom control or quality of life. ICU admission type: Elective = planned ICU admission; Emergency = unplanned ICU admission. Postoperative = admission following surgery; Sepsis / Infection = admission due to infection. Oncologic therapy complication = complications related to cancer treatments. Other = includes causes such as bleeding or airway obstruction. No statistically significant differences were observed between groups.

Supplementary Table S2. Protein Supplementation by Group with p-value

Protein supplements, n (%)	Usual Dose (n=37)	High Protein (n=80)	Total (N=117)	p-value
Total protein supplements	24 (64.9%)	68 (85.0%)	92 (78.6%)	0.011
IV amino acids	0 (0.0%)	13 (19.1%)	13 (14.1%)	
Casein	9 (37.5%)	8 (11.8%)	17 (18.5%)	
Whey protein	21 (52.5%)	47 (69.1%)	68 (63.9%)	

Legend: n (%) = number and percentage of patients in each group. Usual Dose = protein intake ≤1.5 g/kg/day; High Protein = protein intake >1.5 g/kg/day. IV amino acids = intravenous amino acid supplementation; Casein and Whey protein refer to types of enteral protein supplements.

Supplementary Table S3. Sensitivity Analyses Using Intention-to-Treat Population

Outcome	High Protein Group (n=57)	Usual Dose Group (n=60)	p-value	Relative risk RR (CI95%)	Absolute Risk Difference (survival)
ICU survival	77%	65%	0.155	0.65 (0.36 to 1.17)	+12.2%
Hospital survival	90%	82%	0.216	0.57 (0.23 to 1.45)	+7.8%
60-day survival	60%	53%	0.447	0.86 (0.57 to 1.31)	+6.3%
Muscle loss at Day 7	-9.3%	-21.7%	0.066	———	———
Hospital length of stay	21 days (15-30)	26 days (18-38)	0.106	———	———

Legend: ICU = Intensive Care Unit. High Protein Group = patients receiving >1.5 g/kg/day of protein; Usual Dose Group = patients receiving ≤1.5 g/kg/day. Muscle loss at Day 7 refers to the percentage change in muscle mass from baseline to day 7. Hospital length of stay is expressed as median (interquartile range). Relative risk (RR) was calculated only for binary outcomes.
